# A divide and conquer approach to maximise deep learning mammography classification accuracies

**DOI:** 10.1371/journal.pone.0280841

**Published:** 2023-05-26

**Authors:** Adam Jaamour, Craig Myles, Ashay Patel, Shuen-Jen Chen, Lewis McMillan, David Harris-Birtill

**Affiliations:** School of Computer Science, University of St Andrews, St Andrews, Fife, United Kingdom; University of Engineering & Technology, Taxila, PAKISTAN

## Abstract

Breast cancer claims 11,400 lives on average every year in the UK, making it one of the deadliest diseases. Mammography is the gold standard for detecting early signs of breast cancer, which can help cure the disease during its early stages. However, incorrect mammography diagnoses are common and may harm patients through unnecessary treatments and operations (or a lack of treatment). Therefore, systems that can learn to detect breast cancer on their own could help reduce the number of incorrect interpretations and missed cases. Various deep learning techniques, which can be used to implement a system that learns how to detect instances of breast cancer in mammograms, are explored throughout this paper. Convolution Neural Networks (CNNs) are used as part of a pipeline based on deep learning techniques. A divide and conquer approach is followed to analyse the effects on performance and efficiency when utilising diverse deep learning techniques such as varying network architectures (VGG19, ResNet50, InceptionV3, DenseNet121, MobileNetV2), class weights, input sizes, image ratios, pre-processing techniques, transfer learning, dropout rates, and types of mammogram projections. This approach serves as a starting point for model development of mammography classification tasks. Practitioners can benefit from this work by using the divide and conquer results to select the most suitable deep learning techniques for their case out-of-the-box, thus reducing the need for extensive exploratory experimentation. Multiple techniques are found to provide accuracy gains relative to a general baseline (VGG19 model using uncropped 512 × 512 pixels input images with a dropout rate of 0.2 and a learning rate of 1 × 10^−3^) on the Curated Breast Imaging Subset of DDSM (CBIS-DDSM) dataset. These techniques involve transfer learning pre-trained ImagetNet weights to a MobileNetV2 architecture, with pre-trained weights from a binarised version of the mini Mammography Image Analysis Society (mini-MIAS) dataset applied to the fully connected layers of the model, coupled with using weights to alleviate class imbalance, and splitting CBIS-DDSM samples between images of masses and calcifications. Using these techniques, a 5.6% gain in accuracy over the baseline model was accomplished. Other deep learning techniques from the divide and conquer approach, such as larger image sizes, do not yield increased accuracies without the use of image pre-processing techniques such as Gaussian filtering, histogram equalisation and input cropping.

## Introduction

Breast cancer is one of the most common forms of cancer amongst women and is the second most common form of cancer in the world with over 2 million new cases recorded in 2018 [1]. Indeed, 55,200 new breast cancer cases are reported every year in the UK, of which an alarming average of 11,400 lead to death [2]. With an average of 20% mortality rate, breast cancer is ranked as one of the deadliest diseases [3].

**Fig 1:**
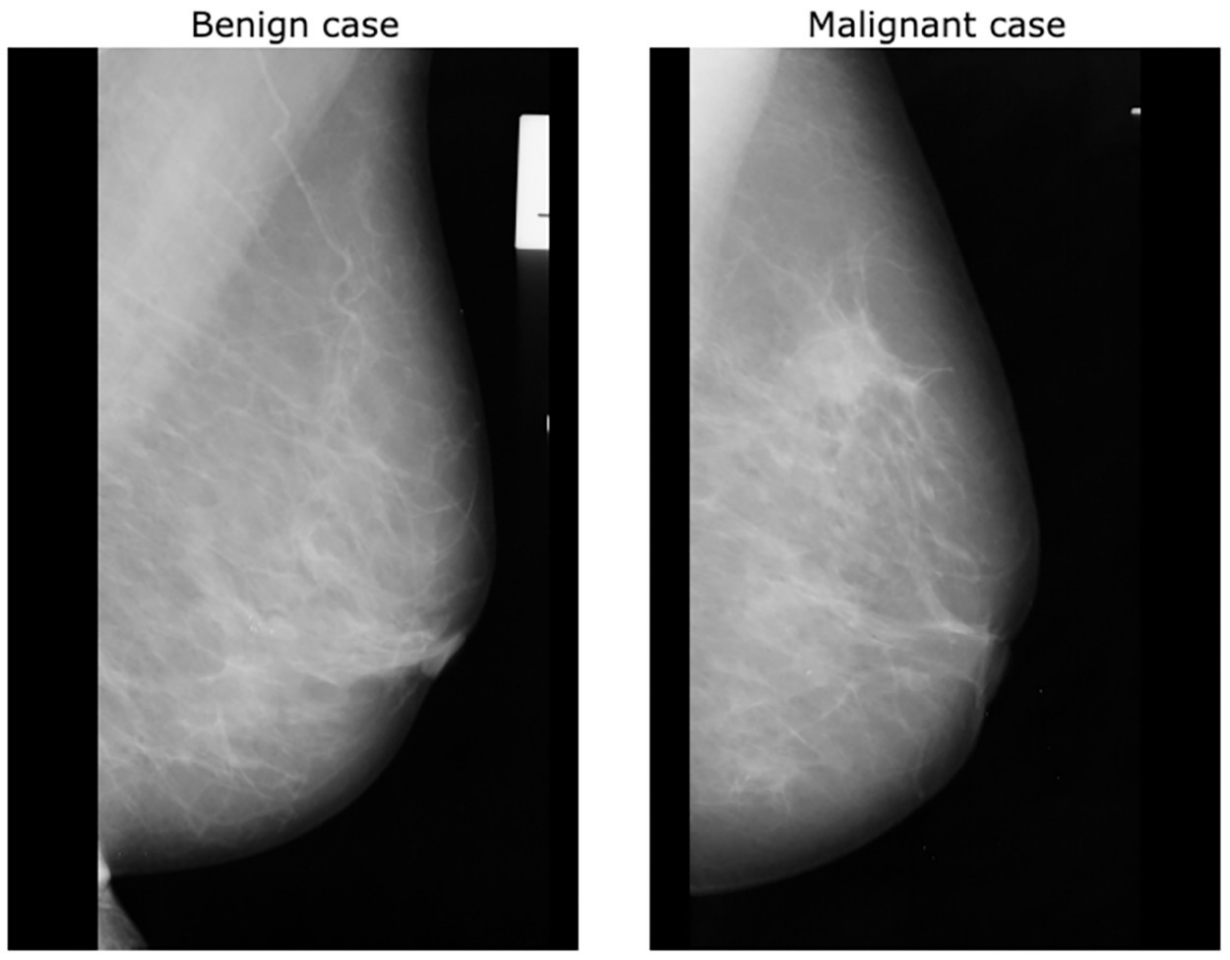
Mammogram labels. Example of benign and malignant cases of mammograms from the mini-MIAS dataset [6].

Early detection of breast cancer through screening tests such as mammograms are an efficient way to maximise patient survival rate by treating the disease prematurely. However, no matter the expertise of radiologists examining mammograms, external factors such as fatigue, distractions and human error need to be minimised [4], as the rate of undetected instances of breast cancer during initial mammogram screenings are as high as 30% [5]. To convey the complexity of mammogram interpretation, Fig 1 illustrates two different mammograms containing benign and malignant samples, revealing how similar they look to an untrained eye. Computer-Aided Detection (CAD) systems using deep learning techniques could, in theory, significantly increase the accuracy of mammography screenings for detecting early signs of breast cancers. However, these techniques require large amounts of data to learn the cancer’s underlying patterns and adapt to new cases, and require powerful computational resources to accelerate the process of learning the data, making them difficult to optimise for.

## Context review

The context review explores the background and existing literature surrounding the techniques used for mammography classification tasks, starting by covering the literature surrounding breast cancer detection before focusing on the use of transfer learning and varying images sizes and regions of interest as input.

### Breast cancer detection context

Test screenings are used to detect early signs of breast cancer before the appearance of any symptoms. The main method used for breast cancer screenings is mammograms, low-dosage x-rays around the breast area [7]. These scans reveal a high contrast background in black with dense areas in whites, which may correspond to calcifications or masses (e.g. lumps or cysts). If suspicious areas are detected, mammograms are followed by breast ultrasounds and MRIs (Magnetic Resonance Imaging) [8]. If any of the screenings raise suspicion or reveal a potential presence of breast cancer, then a breast biopsy, the removal of a small section of breast tissue to be analysed by a pathologist, can be conducted to confirm the screening tests’ results [9]. Due to the invasive nature of biopsies, it is ideal for doctors or clinicians to use medical imagery tools to detect early signs of breast cancer that can be treated early. Despite being the primary imagery method used, mammograms rely on the conventional diagnoses of expert radiologists [10]. These diagnoses rest on the correct interpretation of the mammograms, which may be subject to errors and interobserver variability due to the difficulty in correctly interpreting them [5, 11].

Supervised machine learning techniques are replacing these expert systems, allowing for hidden patterns in mammography data, which can not be perceived by radiologists, to now be recognised by these new algorithms [12]. Machine learning-based approaches were selected over statistical approaches, especially when dealing with large, complex and high-dimensional data like mammography datasets [13]. However, these machine learning models such as k-Nearest Neighbours (kNN) [14], decision trees, Support-Vector Machines (SVM) [7] and Artificial Neural Networks (ANN) [13] can not accurately operate on raw data as they required relevant features ranging from visual information (colours, edges, corners, shapes and textures [15]) to extracted information (cell size, clump thickness, bare nuclei, etc. [13]) to first be engineered from the images. Deep learning models, which corresponds to neural networks with hundreds of hidden layers, are based on the concept that these features can be learned by models on their own, directly from the data [16]. However, these deep models have not been broadly implemented until recent years as they require high powered Graphical Processing Units (GPU) to be trained effectively [17, 18].

### Deep learning applications with transfer learning

Due to recent advances in deep learning techniques, this method of image analysis has shown promise in being transferred to the medical image domain, and more specifically for use in disease classification, detection and segmentation.

Given the size of deep learning networks, training an entire model from scratch is an intimidating task which requires huge amounts of data, time and compute resources to achieve accurate results [19]. To alleviate this issue, transfer learning is often utilised. This involves using networks which are pre-trained on large yet different datasets, and using these pre-trained weights as a starting point for further training on the desired dataset for the specific task, effectively fine-tuning the model [20]. The aim of this is to improve a network designed for a specific domain by transferring that which has already been learnt from another similar domain [21]. An analogy used [22] helps to explain this in real life; If one was to compare the ability of an experienced guitarist and someone with no musical experience to learn the piano, the guitarist would learn the piano in a more efficient way through using prior knowledge. This is much like how a network uses its knowledge from previous training as a starting point for learning similar tasks.

The need for transfer learning comes when there is insufficient training data, whether this is due to expense, scarcity or inability to generate labelled data. This is a common problem in the medical imaging domain and more specifically for mammograms as there are very few public databases which exists that contain an extensive number of cases with labelled data. One of the largest public databases, the Digital Database for Screening Mammography (DDSM) [23] has as few as 2600 cases. Second to this, released in April 2021, is The Chinese Mammography Database (CMMD) [24] which contains 3728 images for a total of 1775 patients. It should be noted that both DDSM and CMMD datasets are available freely for research usage via The Cancer Imaging Archive [25]. Papers focusing on the DDSM dataset tend to use complex pipelines consisting of extensive pre-processing procedures. A prominent example is Mohiyuddin et al. who achieved a record accuracy of 96.50% [26] with the use of state-of-the-art algorithms such as YOLOv5 [27]. However, in their work, they design their experiments such that macro calcification are omitted completely and only masses are considered, whereas this study considers both masses and calcifications both together and separately. Another paper with a complex pipeline consisting of several pre-processing, segmentation, false-positive reduction and classification steps, achieved an accuracy of 90.44% and an AUC of 90% [28].

Aboutalib et al. [29] explored the use of transfer learning through an incremental approach for a 3-class classification task on mammograms for negative, benign and malignant cases. The study used an AlexNet [30] architecture. This model made use of transfer learning by using ImageNet for pre-training and then further learning on the DDSM database (9648 images) before finally fine-tuning the model on the Full-Field Digital Mammography (FFDM) dataset (5212 images), a private dataset [29]. To explore the effects of transfer learning, and given that the DDSM database was larger than the FFDM database, Aboutalib et al. compared a model with further pre-training on the DDSM database before fine-tuning on the FFDM dataset against a model with only fine-tuning on the FFDM dataset (note, both models were pre-trained on the ImageNet database and testing was done on the FFDM dataset). It was observed that the effects of further pre-training on the DDSM dataset helped to improve the model’s Area Under the Curve (AUC) performance by an average of 3.2% across the 6 classification pairs. That being said the final performance only managed to generate an AUC of 76% and the results showed the model had a clear weakness in diagnosing malignant cases as opposed to benign. This points towards the fact that there are imaging features unique to the recalled benign cases which the network is able to successfully identify, features that are much harder to pick up for the malignant cases where the network is less successful.

### Comparing full image against regions of interest as model input

The lack of accuracy generated by some models and deep learning techniques can often be traced back to the size of the image and the corresponding Regions of Interest (ROI) where breast masses and calcifications occur. The ROI can often vary in size and be a very small proportion of the entire image. As a result, this makes it more difficult for diagnosis [31]. A common setup in research has involved using pre-annotated ROIs for classification purposes. This approach was taken by Chun-ming et al. [32], in undertaking a 5-class classification task: benign calcification, malignant calcification, benign mass, malignant mass and a normal breast. The paper explores the creation of a Deep Cooperation Neural Network consisting of two parallel CNNs that feed into a fully connected network. The research made use of data from the DDSM archive, where pre-annotated ROIs were used for images with calcifications and masses, and cropped images of the same size were used for normal breast images, all with image sizes of 299×299 pixels. For a negative-class classification task, this network was able to achieve an accuracy of 91% with an AUC of 98%, a drastic improvement from the full mammogram classification carried out by Aboutalib et al, emphasising the advantage of using cropped images. Similarly, Levy et al. [33] used a similar approach in determining the malignancy of masses using the DDSM database. This research involved a comparison of three CNNs, a baseline shallow CNN with three convolutional layers and three fully connected layers along with AlexNet and GoogLeNet [34]. As before, pre-annotated ROIs were used for the classification task, with padding equal to 50 pixels (small context) and padding equal to a given amount such that the ROI equals to twice the size of the mass bounding box (large context). The results showed that, by utilising the large context enhanced classification method, accuracy with the GoogLeNet CNN architecture reached 93%. From this research, however, it is apparent that the accuracy of using CNNs for breast cancer classification is improved by using more specific ROIs, for a closer view of tumours, in order to generate more accurate results. This is an important point as it is a critical idea that can aid classification accuracy, a primary objective of this research paper. The issue with this however is similar to the issue represented by using ANNs, SVMs or kNN algorithms for classification using pre-extracted features, in that, image pre-processing and ROI tagging is required for an accurate prediction. This is then again reliant on trained radiologists generating ROIs to be used as an input to a CNN for breast cancer detection. Thus, bringing back the initial issues of being reliant on human input.

The inherent difficulties and limitations associated with classification of breast tumours using whole images alone leads onto the next wave of research based on automated extraction of ROIs and/or feature extraction for cancer classification using whole images. The effectiveness of using ROIs is clear; given the size of most tumours in respect to the entire image, a large majority of the image is essentially background noise that can throw off a classifier which is why it is important for a classifier to be able to learn and recognise the ROIs for classification tasks. This is achieved in multiple ways, from ROI tagging to mass segmentation and classification. An example of this is carried out by Punitha et al. [35]. In this research, an optimised region growing technique is used for breast mass segmentation. The segmentation algorithm uses a thresholding technique for region growing with thresholds and optimised seed points. Once segmented, GLCM and GLRLM (Gray Level Co-occurrence Matrix and Gray Level Run-Length Matrix) feature texture extraction is carried out on the mass. The results are then fed into a simple feed-forward neural network for classification. Through the use of segmentation and feature extraction, the classifier was able to achieve a sensitivity and specificity of 98.1% and 97.8% respectively and the final classification task had an accuracy of 98%. This process has effectively brought forward the strengths of using prior machine learning classifiers with high accuracy used on extracted features whilst managing to automate the feature generation through segmentation and automatic feature extraction. In doing so, research such as this highlights the importance of being able to detect where a mass or calcification is, either via bounding boxes or segmentation, in order to enhance classification tasks without the need for prior human input.

However, the implementation of multi-stage detection systems can add greater degrees of error into the system. Although these may appear to have high accuracy, the drawback of such a system is that the entire classification becomes increasingly dependent on the accuracy of the earlier detection stages. If, for example, one uses a mass segmentation system followed by a classification of the segmentation for cancer detection, this firstly relies on the full successful segmentation of a tumour followed by the successful classification of that tumour. As such, it can be seen why there is value in a single stage whole image classification system that has no prior reliance on other levels of image analysis.

The literature covered in this context review analysed the various techniques already used in mammography classification, ranging from machine learning and deep learning techniques to pre-processing steps. Early breast cancer detection methods such as expert systems and supervised machine learning-based techniques, lacked the performance attainable from deep learning techniques. Recent datasets have been beneficial in medical imaging, such as CBIS-DDSM for mammography classification. However, most studies using these datasets have made use of complex pre-processing techniques to obtain improved performance. Unfortunately, this makes their models reliant on tailored pre-processing pipelines to extract this performance and can reduce model generalisability. Alternatively, this study concentrates on these deep learning techniques and the performance gained by combining them to maximise classification accuracy instead. Accordingly, the divide and conquer approach proposed in the Materials and Methods section focuses on achieving that, rather than focusing on a single model and relying on optimised data that is pre-processed.

## Materials and methods

This section covers practical aspects of the paper, including reproducibility, resources used, ethics statements, datasets used, and the techniques covered in the divide and conquer approach (data preparation, model training and model evaluation).

### Reproducibility

To maximise reproducibility, the code used to implement the deep learning techniques in this paper, as well as the instructions to download the relevant data and run the pipeline, have all been made open-source and can be accessed online at https://doi.org/10.5281/zenodo.6828154.

### Resources

Computational resources, in the form of a GeForce GTX 1060 6Gb GPU, were used to train models. To make use of the GPU’s compute capabilities, Tensorflow [36] and Keras [37] were used. These provide deep learning frameworks with CUDA support (to enable GPU optimisation and parallelisation), CNN support and pre-trained models.

### Ethics statement

Datasets utilised in this paper include CBIS-DDSM and mini-MIAS, both of which are publicly available with all personal data anonymised and removed by default. In addition to the mammography images, both datasets supply accompanying diagnostic information. This accompanying data does not include any identifiable labels. Additionally, this study was approved by the University of St Andrews School of Computer Science Ethics Committee on behalf of the University Teaching and Research Ethics Committee (UTREC). Both datasets were not created by the authors and no participants were involved in this study. Therefore, no further consent-taking or ethics approval was needed.

### Data

The deep learning models implemented in this work require clinical data to learn the underlying patterns necessary to detect cases of breast cancer in mammograms and to evaluate its performance. Therefore, the CBIS-DDSM and mini-MIAS datasets, fully anonymised, open-source and public datasets, were used.

The Curated Breast Imaging Subset of Digital Database for Screening Mammography (CBIS-DDSM) dataset, an open-sourced, anonymised public dataset [38], is available online from The Cancer Imaging Archive [25]. The dataset, which contains a total of 10,239 images in Digital Imaging and Communications in Medicine (DICOM) format gathered from 1,566 patients across 6,775 studies [39], is the main dataset used in this work. This dataset is an updated and standardised subset of the older DDSM dataset [40], containing only abnormal cases with benign and malignant tumours (no normal cases). The scans found in the dataset are a mix of the two most commonly used projections in routine mammogram X-ray scans: bilateral craniocaudal (CC) and mediolateral oblique (MLO) [5]. The dataset can be further be separated into two different types of structures that radiologists usually look for to detect early signs of breast cancer: calcifications (small flecks of calcium usually clustered together) and masses (e.g. cysts or lumps) [41].

The mini Mammography Image Analysis Society dataset (mini-MIAS) [6] is a smaller open-sourced, anonymised public dataset available online from Pilot European Image Processing Archive (PEIPA). It is used solely for transfer learning purposes. It contains 322 images in greyscale Portable Gray Map (PGM) format with associated ground truth data and images all reduced to a uniform size of 1024×1024 pixels [42]. It is divided into normal, benign and malignant cases [43], but only the benign and malignant cases were used in this work.

**Fig 2:**
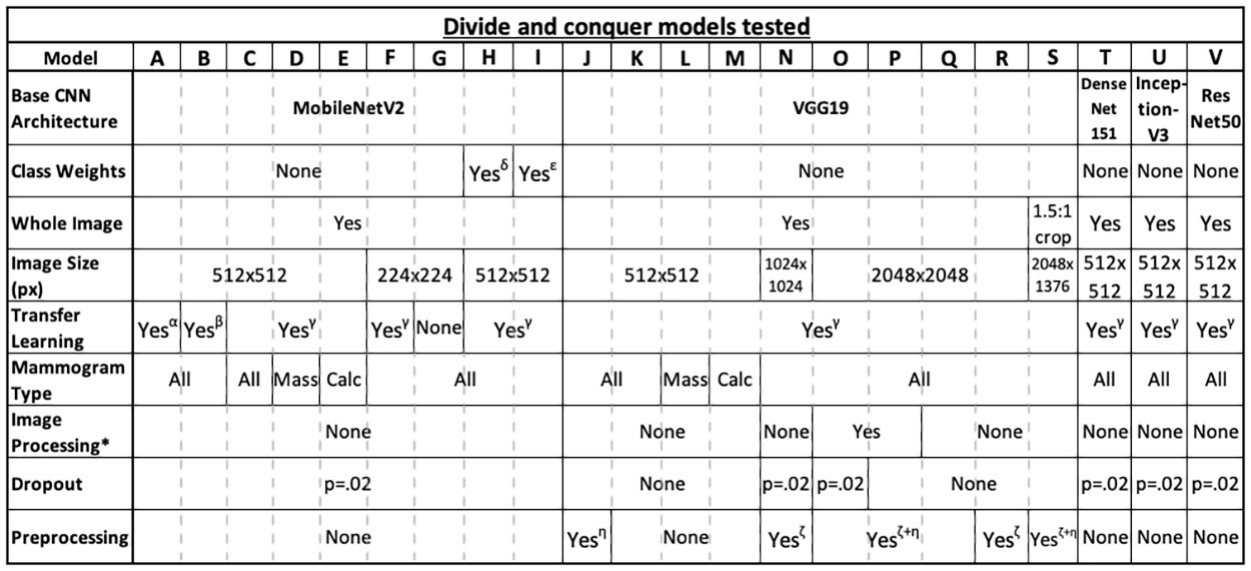
Divide and conquer experiments summary table. Table comparing the different divide and conquer models tested using various deep learning techniques covered in the Materials and Methods section. * Histogram equalisation + Gaussian filtering. *α*: All layers instantiated with pre-trained weights from a binarised mini-MIAS dataset. *β*: Base CNN layers instantiated with ImageNet weights; fully-connected layers instantiated with pre-trained weights from a binarised mini-MIAS dataset. *γ*: Base CNN layers instantiated with ImageNet weights; fully-connected layers instantiated with random weights. *δ*: Balanced class weights (0.907 for majority benign class, 1.113 for minority malignant class). *ϵ*: +50% class weight for minority class (1.0 for majority benign class, 1.5 for minority malignant class). *ζ*: Two sets of convolutional layers before base CNN. *η*: Convolutional layers before fully-connected layers.

The methods outlined in this section are implemented using a divide and conquer approach, whererin a combination of techniques are iteratively tested and evaluated to build an understanding of which perform best. They include a mixture of data preparation and model training techniques, combined as shown in Fig 2.

### Data preparation techniques

#### Dataset balance

**Fig 3:**
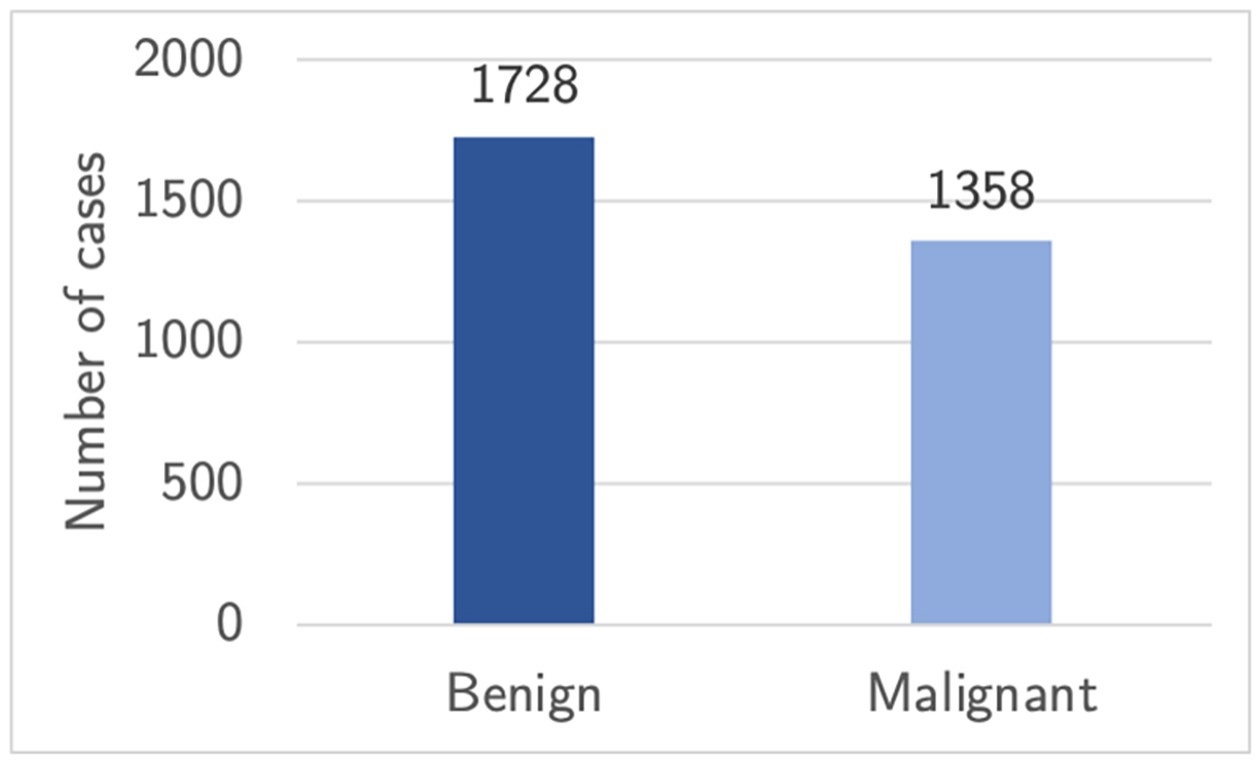
Dataset imbalance. Bar chart illustrating the imbalance of classes in the CBIS-DDSM dataset.

The class distributions seen in Fig 3 reveal that the CBIS-DDSM dataset is imbalanced, which must be taken into account to avoid fitting a biased CNN model. Potential solutions for countering this imbalance are to either undersample the majority class or oversample the minority class. Undersampling the dataset can be considered inefficient as it will diminish the number of samples the model could learn from [44]. However, oversampling by creating new artificial images resembling the original data, referred to as data augmentation, is a viable solution as it was proven to decrease the risk of overfitting [45]. Alternatively, a cheaper option in terms of computing resources would be to add class weights, which will cause the loss to become a weighted average giving more importance to less frequent classes [46].

#### Dataset preparation

Multiple steps were followed to ensure that the data was in an optimal format before being used as model input. The dataset did not need to be manually split as it was already pre-split with a 80%/20% ratio when it was originally created [39]. These splits were stratified to maintain representative samples from the data in both the training and the testing sets to avoid introducing sampling bias. Containing 10,239 images, which equates to 163.6 GB of disk space, the dataset cannot be loaded in memory in a single import and needs to be loaded in batches to be fed into the CNN sequentially. Additionally, the mammography images were resized to target sizes, specified in Fig 2, during import to scale them down (as original images are larger than 3000×5000 pixels) and to avoid having inconsistent input sizes.

Because the weights in neural networks are usually initialised with values between 0 and 1, utilising large input values for the pixel intensities ranging from 0 to 255 can disrupt and slow down the fitting process, ultimately leading to lower performances. Therefore, normalising the pixel intensities to values in the range of 0 to 1 can solve this problem by ensuring all values are small enough to reduce computational workload.

Finally, as the labels for each mammogram are in categorical string format, they must be encoded into a numerical format. To do so, binary encoding is used, labelling ‘benign’ and ‘malignant’ cases as *0* and *1* respectively.

#### Image enhancement

A number of image processing steps were taken as optional augmentations to aid network performance. These were:

- Optimised image cropping
- Gaussian filtering
- Histogram equalisation

The first step was to use optimised cropping dimensions. From inspecting the mammography images, it was clear that image sizes ranged from roughly 2000×4000 to 3000×5000 pixels. In this work, VGG19 and DenseNet, models that are originally designed for square images of size 224×224 pixels, were used, which was taken into account for the initial sizes. It was however decided that because sizes of 224×224 pixels would require significant downsizing, hence the model input was scaled up to 512x512 pixels, and a square image ratio was preserved.

**Fig 4:**
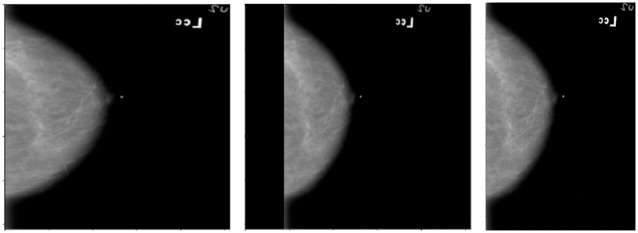
Image resizing and padding. Demonstration of varying resizing and padding of the same image: Resized to fill image without padding—aspect ratio not preserved (left), resized to fill image with padding—aspect ratio preserved (middle), resized and cropped without padding—aspect ratio preserved (right).

There are two ways an image can be resized: resizing it such that the image takes up the entire dimensions, i.e. stretch or compress it to a desired dimension, or resizing the image with padding to maintain the image’s aspect ratio. Although the images were initially scaled to a square size without padding, it was determined that this would distort the images’ spatial features through expansion along the horizontal axis. Accordingly, resizing with padding was used to maintain aspect ratios. The issue, however, was that given the usual dimensions of the image, by resizing with padding, a large amount of the image would become blank space. In order to reduce this, the best option was to change the input size to reflect the natural aspect ratio of the mammogram images. In general this aspect ratio was around 1.5:1. Choosing input dimensions that conformed to this level resulted in much less black space in the images as compared to the square images, whilst preserving the natural aspect ratio. An example of the varying resizing of the same image can be seen in Fig 4 below.

Additionally, it has been shown that the use of Gaussian filtering and histogram equalisation can enhance the details in mammograms, to optimise them for use with CNNs. As implemented by George et al. [47], a Gaussian filter with *σ* = 0.5 was applied, along with histogram equalisation as applied by Cheng et al [48]. A Gaussian filter is a 2D convolutional filter that is used to blur an image and remove detail. In general, depending on the value of *σ*, a large blur is not used since vital details will be lost. However, this filter is used to remove noise inherent to the image itself.

**Fig 5:**
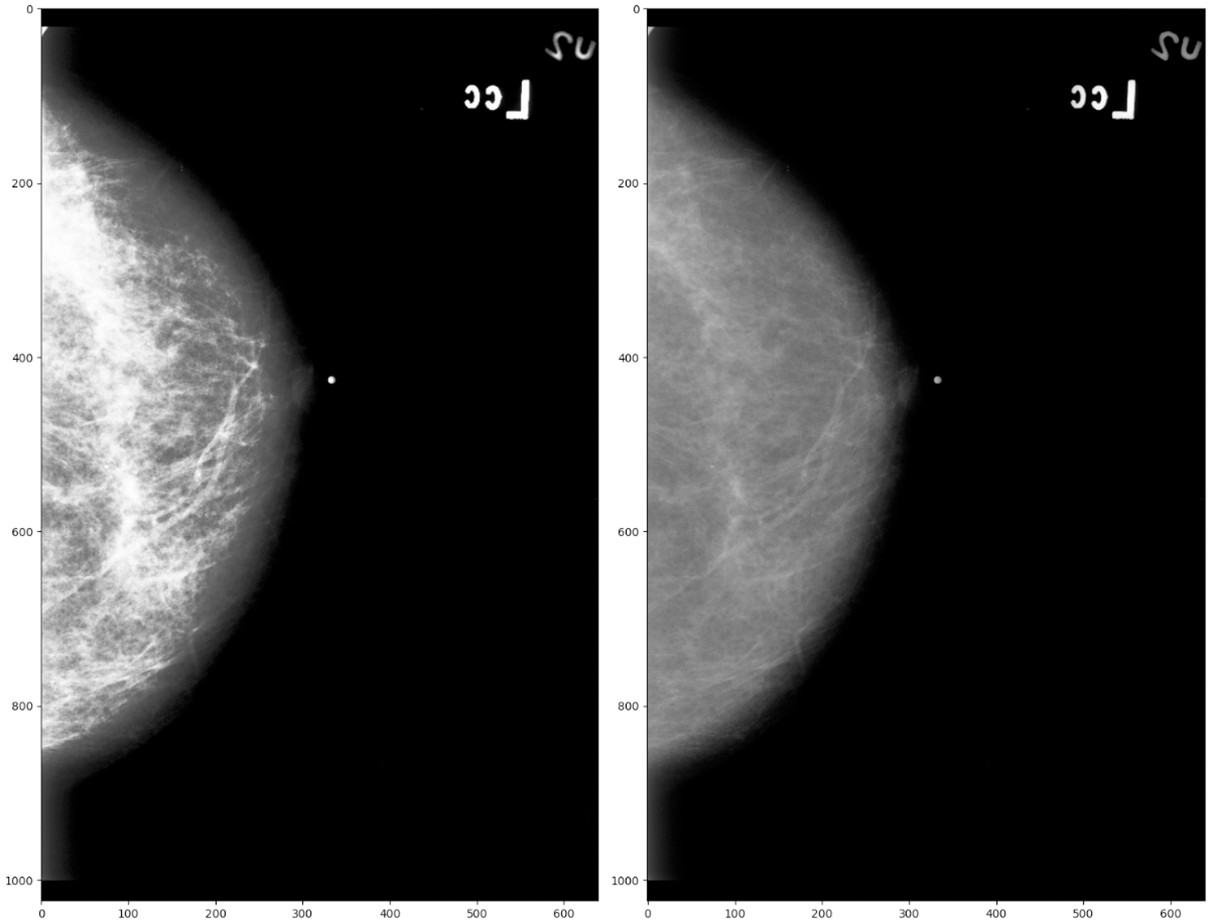
Image enhancements. Mammogram scans with (left) and without (right) histogram equalisation and Gaussian filtering.

Histogram equalisation is an image processing technique used to improve the contrast in an image. It does this by spreading out the most frequent intensity values and stretching them over the intensity range. The combination of Gaussian filtering and histogram equalisation is used as another pre-processing step to enhance the quality of the image input. This step can be visualised in Fig 5.

### Model training techniques

At this stage of the pipeline, the training data is ready to be fed into the CNN model, where it will learn from the images that were processed using the steps above, before making predictions on unseen test dataset, which will be compared with the ground truth labels for evaluation.

#### CNN model

Due to the very small nature of the CBIS-DDSM dataset (approximately 3000 samples), the knowledge of CNN models pre-trained on large general datasets such as ImageNet (which contains over 14 million images belonging to 22000 categories [30]) is used via transfer learning, a technique that is proven to work on breast cancer detection tasks [49, 50]. This leads a general CNN to converge towards one that is tailored for the task of breast cancer detection using mammography images, rather than creating one from scratch. Different CNN architectures can be used as the base of a custom CNN model tailored for breast cancer detection. This is achieved by using popular CNN architectures natively available within Keras such as VGG19 [51], ResNet50 [52], InceptionV3 [53], DenseNet121 [54] and MobileNetV2 [55] as the base of the CNN [56]. The fully connected layers of these models, originally designed for general classification of 1,000 different categories, were dropped from the model and replaced with a custom MLP (Multi-Layer Perceptron) [57]. The fully connected MLP contains hidden layers and an output layer with different activation functions based on the dataset used.

**Fig 6:**
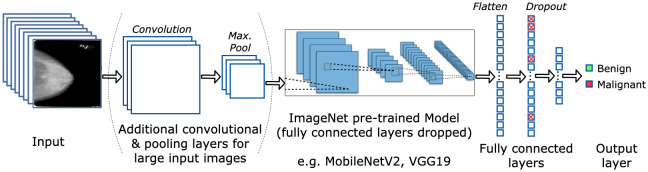
Model architecture. Model architecture implementation, which includes input images, additional convolutional and max pooling layers, ImageNet pre-trained convolutional layers (e.g., on either VGG19 or MobileNetV2 architectures), fully connected layers (flatten, dropout) and the output layer. Original image, “ImageNet pre-trained model” by Aphex34 [59].

Depending on the size of the image used, additional convolutional and pooling layers were added before the base model to downsample the image to smaller sizes and learn lower-level features. This was followed by the pre-trained base model, a flatten layer to convert the output from 2D to 1D, and finally the MLP which will make the final prediction. Dropout layers were added in the MLP to avoid overfitting [58]. The full model can be visualised in Fig 6.

#### Model training

Different activation functions can be used in the output layer (final layer) of the model. Because this is a binary classification problem, a single neuron with a sigmoid activation function is used as it outputs an independent value between 0 and 1 that can be interpreted as a probability of the positive class.

Cross entropy is one of the most commonly used loss functions as it can be used for any classification task that estimates probabilities [12]. Because the sigmoid function outputs probabilities, cross entropy is the ideal loss function as it heavily penalises the model when a low probability is predicted for the target class [20].

Due to the complex nature of the model, it is important to minimise the number of optimisable hyperparameters. Adaptive learning rate algorithms usually generalise better than traditional optimisers [60] such as Stochastic Gradient Descent (SGD) [61] or momentum, which are slow to converge and require more fine-tuning. The most general adaptive optimiser is *Adaptive moment estimation* (Adam) [62], which combines both momentum for more significant steps in the direction of the steepest gradient and Root Mean Square Prop (RMSProp) for more accelerating on steep slopes than small slopes, making it the best choice for this model.

To best make use of the transfer learning technique with the base model’s weights instantiated with ImageNet weights, training was separated into two phases. In the first phase, all the layers from the base model were frozen, enabling only the custom MLP with fully connected layers to fit the mammogram images. The initial training phase ends once the maximum number of epochs is reached, or the early stopping condition is met. In the second phase, all the layers were unfrozen, and training was resumed with a lower learning rate of 1 × 10^−5^, allowing the base model to slightly alter its weights to adapt to the mammogram dataset while not forgetting the previously learned knowledge from the ImageNet weights.

To ensure that the model generalises well to the unseen data from the testing set, the training set is further split to form a validation set using a 75%/25% split, resulting in a respective 60%/20%/20% train/validate/test split of the original dataset. The validation set is used to make predictions at the end of each epoch by calculating the loss and accuracy on it. The loss on the validation set is monitored against the number of epochs to determine whether to stop the training earlier than a preset maximum number of epochs in order to prevent the model from overfitting the data if the loss does not improve as the number of epochs increases. An alternative technique to using a validation set, *K*-fold cross-validation, which divides the training set into *K* subsets and evaluates the model *K* times to avoid overfitting, was not used due to time constraints. Since fitting the model on the CBIS-DDSM dataset takes between 1h15m-8h49, this would be multiplied by a factor of *K* when using cross-validation, hence leading to the usage of a validation set instead.

#### Hyper-parameter optimisation

**Fig 7:**
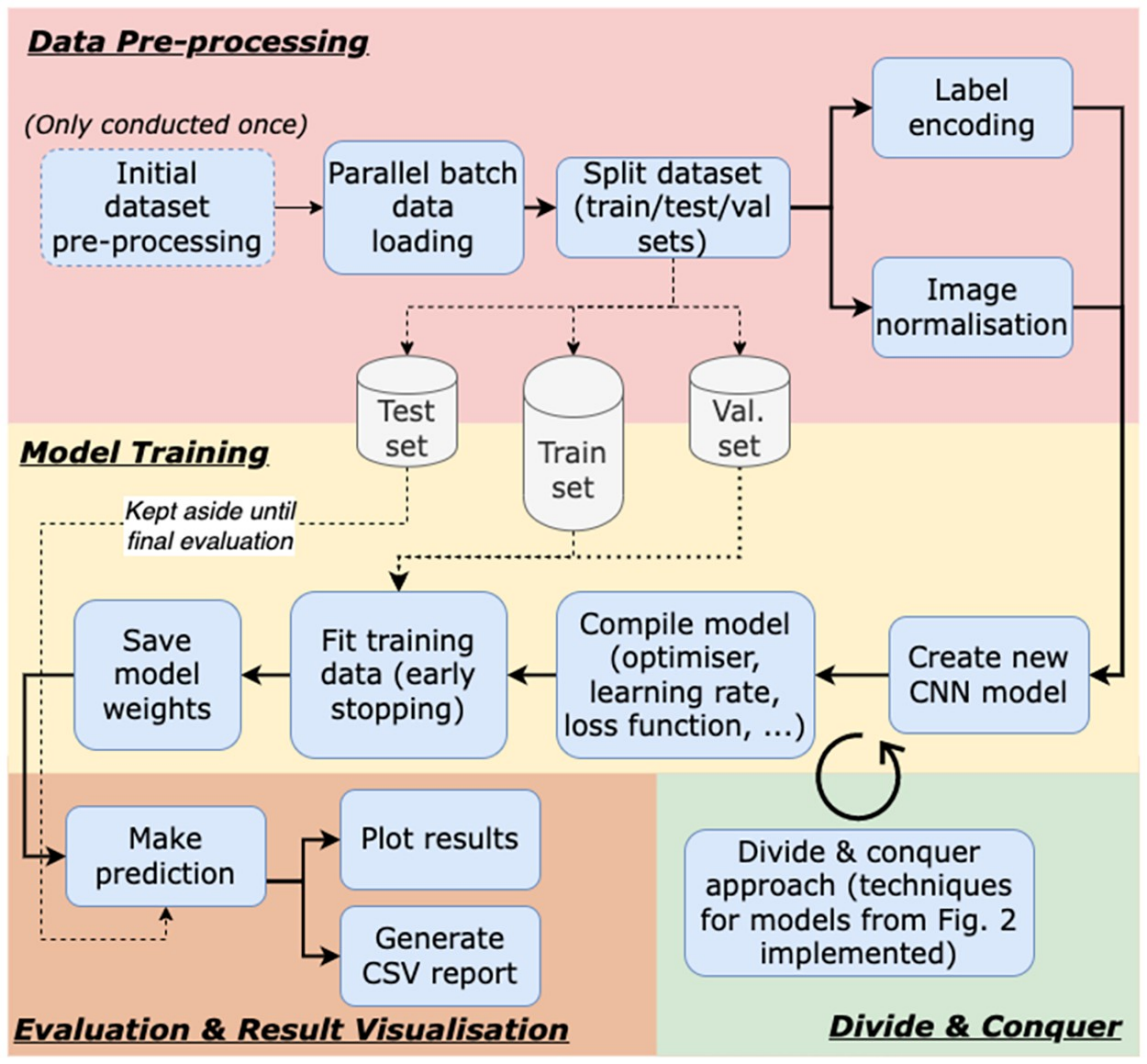
Flowchart pipeline. A detailed flowchart of the implemented breast cancer detection deep learning pipeline.

Traditionally, a grid search approach would have been preferred to fine-tune the model’s hyperparameters. However, due to the project’s time constraints, the significant training runtime and the number of hyperparameters to fine-tune, a grid search would have been an unrealistic approach. Instead, a divide and conquer approach was selected, manually implementing and validating different combinations of deep learning techniques mentioned in this section. The combinations tested are summarised in Fig 2, and include using different pre-trained CNN models, variations in transfer learning, data augmentation, dropout values and input image sizes. The methods mentioned across this section and the pipeline proposed are summarised in Fig 7, covering the data preparation steps followed, the model training steps, and the model validation.

### Model evaluation metrics

In mammography classification, overall accuracy (see Eq 1, [49]) is often complemented with additional metrics to evaluate how well the classifier fitted the data. The following terminology is used to define the metrics that are broken down below:

- *P*: Positives (all positive predictions).
- *N*: Negatives (all negative predictions).
- *TP*: True Positives (positive case correctly predicted as positive).
- *TN*: True Negatives (negative case correctly predicted as negative).
- *FP*: False Positives (negative case incorrectly predicted as positive).
- *FN*: False Negatives (positive case incorrectly predicted as negative).

Detecting FPs and FNs is essential to avoid interpreting malignant tumours as benign and vice versa. Such an interpretation could harm the patient and potentially lead to their death. Therefore, a mixture of additional metrics were used to assess how well the model learns the mammograms data and generalises to unseen cases.

Precision, which corresponds to the number of correct positive predictions (see Eq 2, [44]), shows the model’s ability to avoid labelling negative instances as positive. Recall, which is the number of positive instances that are correctly predicted (see Eq 3, [44]), shows how well the model can find all positive instances. Together, they can be combined into a more concise metric, the *F1 score* (see Eq 4, [20]). To achieve a high F1 score, both precision and recall must be high (unlike a regular mean) because as the precision goes down, the recall goes up, and vice versa, making the F1 score a reliable metric for evaluating a classifier since a balance between precision and recall must be found [20].

$$\begin{array}{c} Accuracy=\frac{TP+TN}{P+N} \end{array}$$ (1)

$$\begin{array}{c} Precision=\frac{TP}{TP+FP} \end{array}$$ (2)

$$\begin{array}{c} Recall=\frac{TP}{TP+FN} \end{array}$$ (3)

$$\begin{array}{c} F_{1}=\frac{2}{\frac{1}{precision}+\frac{1}{recall}}=\frac{TP}{TP+\frac{FN+FP}{2}} \end{array}$$ (4)

### Pipeline generalisation

This workflow can be generalised to other breast cancer detection mammography classification tasks as it can be applied to any dataset similar in nature, and could also be used as a starting point for comparable medical imaging tasks such as MRI classification. To evaluate the generalisability of the model across a larger range of demographics and medical imagery techniques, future work would be needed to test the model’s performance on other mammography datasets acquired from other geographical locations and medical devices. Additionally, all of the code implemented for the steps in this Materials And Methods section has been made publicly available and is hosted on GitHub for reproducibility [63].

The different techniques covered in this section are all tested iteratively in a divide and conquer fashion. The results of each experiment from Fig 2 are presented and discussed in the following sections.

## Results

The methods described in the previous section serve as the baseline model for all divide and conquer techniques tested, as it uses the most general and adaptive settings for a CNN. This will allow for a clear depiction of whether a technique will help to increase the performance in the context of mammography classification for breast cancer detection. This baseline model has the following specification:

- VGG19 base CNN model.
- Whole images with sizes of 512×512 pixels.
- Dropout rate of *p* = 0.2.
- Adam optimiser using a learning rate of 1 × 10^−3^.

There are a number of varying factors that are tested and discussed in the next section using a divide and conquer approach. These include tuning the base CNN architecture, batch size, class weights, data augmentation factor, dropout rate, input size, weight initialisation, type of mammograms, use of additional convolutional layers, use of whole or cropped images and image pre-processing techniques. A few parameters remain constant throughout the experiments, including the structure of the fully connected layers (512 and 32 hidden neurons and 2 output neurons), as well as the optimiser used (Adam optimiser with a learning rate of 1 × 10^−3^ when using VGG19 and 1 × 10^−4^ when using MobileNetV2).

**Fig 8:**
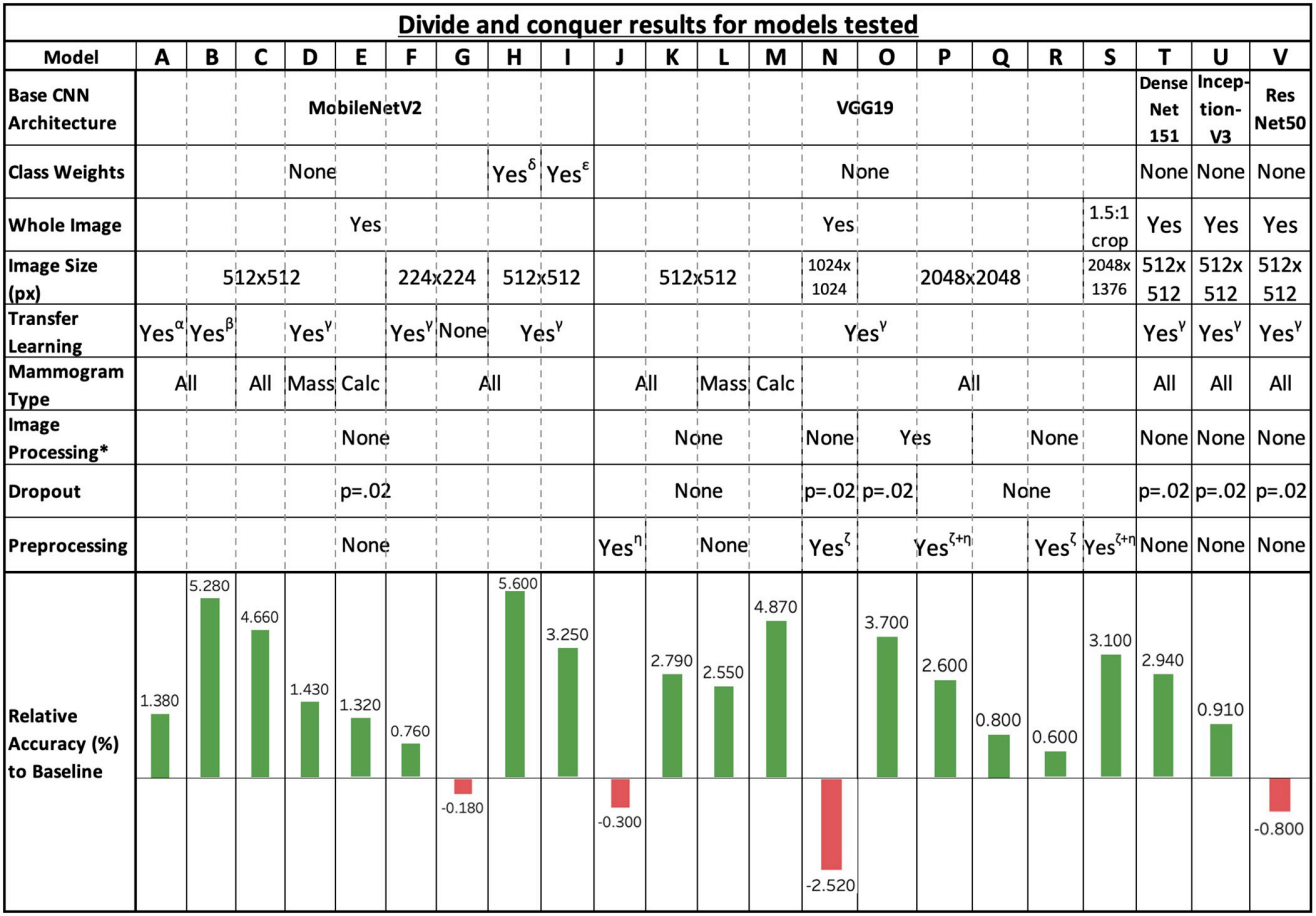
Divide and conquer results summary table. Bar chart comparing the results achieved using various deep learning techniques relative to the baseline (see Results section for baseline implementation specification). * Histogram equalisation + Gaussian filtering. *α*: All layers instantiated with pre-trained weights from a binarised mini-MIAS dataset. *β*: Base CNN layers instantiated with ImageNet weights; fully-connected layers instantiated with pre-trained weights from a binarised mini-MIAS dataset. *γ*: Base CNN layers instantiated with ImageNet weights; fully-connected layers instantiated with random weights. *δ*: Balanced class weights (0.907 for majority benign class, 1.113 for minority malignant class). *ϵ*: +50% class weight for minority class (1.0 for majority class, 1.5 for minority class). *ζ*: Two sets of convolutional layers before base CNN. *η*: Convolutional layers before fully-connected layers.

The CBIS-DDSM test dataset contains 641 test samples, of which 381 are benign and 260 malignant. With the baseline model, as described above, an overall accuracy of 61.80% is achieved on the CBIS-DDSM predefined test set. This metric is used as the relative benchmark that is compared to the results obtained through the various explored techniques. All future references to model names (e.g., “model X”) represent one of the divide and conquer experiments found in Fig 8, where X ranges from A to V.

## Discussion

Using the accuracy of the baseline model defined in the previous section, the combinations of deep learning techniques covered throughout the previous sections are discussed in this section to serve as frame of reference for mammography classification tasks.

### Base CNN architectures

Five different CNN architectures, pre-trained on ImageNet (VGG19, ResNet50, InceptionV3, DenseNet121 and MobileNetV2), are tested out as the base architecture of the model. For this test, the CBIS-DDSM dataset is used with whole images resized to 512×512 pixels, a batch size of 2 and a learning rate of 1 × 10^−4^.

The results clearly reveal that MobileNetV2 outperforms the other CNN architectures, achieving a higher accuracy and F1 score. The baseline model’s VGG19 architecture is outperformed by more efficient models such as DenseNet121 (model T) and MobileNetV2 (model C), while still performing better than both the models that use ResNet50 (model V) and InceptionV3 (model U) as base architectures. These results contradict Falconi’s results on the CBIS-DDSM dataset, which find that ResNet50 outperforms MobileNetV2 [50]. However, MobileNetV2 still outperforms InceptionV3. These results may differ due to the different pre-processing techniques being used as Falconi et al. uses cropped images around ROIs, whereas whole images are used in this experiment. It is also worth noting that using MobileNetV2 as base architecture already surpasses the baseline by 4.66%, and beats models that use traditional machine learning methods like SVMs with GLCM features (63.03% accuracy) on the CBIS-DDSM dataset [64].

However, observing the training and testing runtimes reveals that VGG19 takes the longest time to train (3h50m), whereas the more efficient MobileNetV2 architecture takes less time (2h46m). Additionally, inference runtime is 2.3 times faster with MobileNetV2 compared to VGG19, which ultimately is more useful, in practice, for clinics as mammogram diagnosis results can be returned faster.

### Class imbalance

Distinct variations of class weights are used on the CBIS-DDSM dataset in attempt to rectify the adverse effects that can be introduced by imbalanced datasets. This method avoids the process of data augmentation, which considerably slows down the training time. Three unique class weights values were tested using the imbalanced CBIS-DDSM dataset with whole images resized to 512×512 pixels, a batch size of 2 and a learning rate of 1 × 10^−4^:

- No class weights (dataset remains imbalanced).
- Balanced class weights:
  - 0.907 for majority class (benign).
  - 1.113 for minority class (malignant).
- Pre-determined +50% class weight for minority class:
  - 1.0 for benign samples.
  - 1.5 for malignant samples.

The results using using balanced weights clearly show a 0.62% increase to the accuracy (model H) relative to the one that uses no class weights (model C), thus helping against the imbalanced dataset issue without the need for techniques like data augmentation. However, the pre-determined weight increase for the minority class decreases the relative accuracy by 1.41% (model I) to that same model that uses no class weights, revealing the complexity and importance of finding the right parameters for balancing datasets since a 50% weight increase for malignant samples introduced even greater imbalance.

### Input image sizes

#### Whole images

Various image sizes were explored to determine their effect on the model’s performance. For the smaller image sizes, larger batch sizes are used, whereas, for the larger image sizes, smaller batch sizes are defined, along with extra convolutional and pooling layers, to accommodate the larger image size:

- 224×224 pixels (chosen as most CNNs pre-trained on ImageNet use this size) with a batch size of 8.
- 512×512 pixels with a batch size of 2.
- 1024×1024 pixels (with an additional set of 2 convolutional and pooling layers) with a batch size of 2.
- 2048×2048 pixels (with an additional two sets of 2 convolutional and pooling layers) with a batch size of 2.

The results clearly outline the accuracy increase when using 512 pixels-wide input size rather than 224 (model F), observing a 2.03% increase with VGG19 (model K) and 4.52% increase with MobileNetV2 (model B) relative to model F. When observing the evolution of the training accuracy and loss when using 1024×1024 pixels input size on VGG19, it can be seen that the validation loss increases while the training loss decreases and that both sets’ training accuracies are increasing as well; which is a typical pattern of a model overfitting the data. Because the model is overfitting the data, a very high precision (66.94%) but low recall (59.28%) can be seen for the 1024×1024 input size. This is highly detrimental since a breast cancer detection system that detects malignant cases as benign could lead a failed diagnosis and, ultimately, the death of the patient.

However, further increasing the input size to 1024 pixels has no positive effect if the images are not pre-processed. Indeed, when not using image pre-processing methods, the accuracy drops by 1.76% on VGG19 (model N) relative to model F and leads to an Out Of Memory (OOM) error on MobileNetV2, despite lowering the batch size to 1. However, using Gaussian filtering and Histogram equalisation with 2048 pixels-wide input size increases the accuracy by 3.70% (model O) relative to the baseline.

As expected, increasing the image size also increases the training runtime, which is worsened further by a factor of 2.4 when adjusting from 224 to 512 pixels, and a factor of 2.8 from 512 to 1024 pixels on VGG19. However, image pre-processing and additional convolutional layers are a necessity to adapt to the larger input size. Nevertheless, the accuracy/training runtime trade-off is not paramount in breast cancer detection as the primary goal is to develop a system that can correctly diagnose early forms of cancers in mammograms as accurately as possible, regardless of the runtime. Ultimately, inference runtimes will play an important role when used in clinics.

#### Cropped images

Through the use of varying image sizes and additional convolutional layers to optimise the baseline VGG19 model, some clear observations can be seen in motivating the use of deeper networks with larger input images. The baseline VGG19 model was designed for smaller images, likely consisting of less obscure features than those found in mammograms. As a result, it achieved a lower accuracy when trained and tested on the CBIS-DDSM dataset. One attempt to improve this was to extend the model with convolutional layers of differing kernel size and filters, in addition to a pooling layers for large image ingestion. This brought about two experiments, the first one for image sizes of 1024×1024 pixels with the additional two convolutional layers and the second one for image sizes of 2048×2048 pixels with a further additional two convolutional layers atop of the previous model. Although small, the latter generated an improved accuracy of 0.80% relative to the baseline (model Q). As suggested, the increase in the number of convolutions allows the network to pick up on finer details that would otherwise go unobserved in the original model, due to the size and resolution of these images.

In a further effort to improve the efficiency of the network, the largest image size was cropped from a square image to a rectangular one of size 2048×1376 pixels, which retained image resolution but partially removed some of the black background and padding present in the scans. This improved sample processing speeds by roughly 20% and saw a 2.9% accuracy increase (model S) relative to the 2048×2048 pixels experiment. This demonstrates that cropping images around a region of interest is less computationally expensive and more accurate. It should be noted this is a rough crop as it is to a fixed size and aspect ratio, and as such, an alternative approach involving more specific cropping right around the breast area of the scan may offer better results.

### Degrees of transfer learning

Instead of using a CNN pre-trained on ImageNet, model weights trained on a binarised version of a small dataset, mini-MIAS, are transferred to the CBIS-DDSM dataset. Four different experiments using identical CNN architectures with variations in transfer learning are tested to assess the effect of transfer learning from the binarised mini-MIAS dataset and ImageNet on the CBIS-DDSM dataset:

- Transfer learning of all layer weights (MobileNetV2 and fully connected MLP layers instantiated with binary mini-MIAS weights).
- Transfer learning of fully connected layer weights (MLP layers instantiated with binary mini-MIAS weights, MobileNetV2 layers instantiated with ImageNet weights).
- Transfer learning of ImageNet weights only (fully connected MLP layers instantiated with random weights, MobileNetV2 layers instantiated with ImageNet weights).
- No transfer learning (MobileNetV2 and MLP fully connected layers instantiated with random weights).

The results clearly indicate that any given form of transfer learning provides an improvement over random weight initialisation (model G). On the other hand, too much transfer learning, by using all the weights from the model trained on the binary mini-MIAS dataset, does not generalise well to the CBIS-DDSM dataset (model A). The best performance of this experiment came from initialising MobileNetV2 with ImageNet weights and the MLP fully connected layers with weights trained on the binarised mini-MIAS dataset (model B), achieving an accuracy increase of 5.28% relative to the baseline, which corresponds to an F1 score of 67.23%. This model was closely outperformed by again using ImageNet weights for MobileNetV2 and random weights for the MLP layers which reached a 5.6% relative accuracy increase (model H). The ImageNet weight transfer method provided a performance gain whilst proving the effective and adaptive nature of CNNs when leveraging knowledge learned from large general datasets on a more specific task like mammography classification. It is worth noting that training is marginally faster when using weights from binary mini-MIAS as the model converges more quickly.

### Mammogram types

To assess how the model would adapt to learning samples from a single type of mammogram, the CBIS-DDSM dataset was separated into *only mass* samples and *only calcification* samples. These results show that the model using VGG19 as a base architecture achieves moderate improvement when mass and calcification samples are separated, reaching a 2.55% accuracy increase (model L) and a 4.87% accuracy increase (model M) relative to the baseline respectively on the test set, while achieving an relative accuracy increase of of 2.79% when using the full CBIS-DDSM dataset (model K). Indeed, all instances are classified as “benign” on the full CBIS-DDSM dataset (either true negatives or false negatives), indicating that the model fails to consistently deal with multiple views and cannot differentiate benign and malignant cases from each other. This outcome is in line with Hepsag’s et al. results, which achieve higher accuracies when classifying either masses or calcifications on another dataset [43], and confirms Elter’s et al. claim that masses are more difficult to detect than calcifications [5].

However, the opposite effect is witnessed when MobileNetV2 is used as a base model, reaching an accuracy increase relative to the baseline of 4.66% (model C) when the full dataset is used and only 1.32% (model E) and 1.43% (model D) for calcifications and masses respectively, contradicting the previous results. Because CNNs automatically learn features, it can be hard to know exactly what goes on underneath the hood of these models, especially when using architectures like VGG19 and MobileNetV2 due to their complex structures. Visualising heatmaps of the feature maps for each convolution layer could help understand why these models react differently when using all images or only specific types of mammograms, but is out of the scope of this work.

### Image pre-processing steps

Following the successful implementation of various models, the next step was to move beyond the model fine-tuning and towards the data itself to improve model performance. This was undertaken through image processing techniques that involved histogram equalisation and Gaussian filtering. Histogram equalisation, in particular, is used to help with contrast adjustment. This can be helpful in highlighting important features by increasing contrasts to further highlight masses and/or calcifications. Through applying these image processing techniques, the data was then used to train and test using the VGG19 architecture with image sizes of 2048×2048 pixels and two additional sets of two convolutional and pooling layers. This generated the greatest improvement in accuracy with an accuracy increase of 1.8% (model P) in comparison to the same model without any image pre-processing steps (model Q). This further emphasises the effectiveness of image pre-processing steps such as Gaussian blur and histogram equalisation for mammography classification tasks.

### Dropout rate

Although early stopping was implemented during training to avoid overfitting, it has been shown that adding dropout layers to the fully connected portion of the model can help eliminate overfitting [58]. Applying dropout layers with a drop rate *p* = 0.2 to the VGG19 architecture with image sizes of 2048×2048 pixels and two additional sets of two convolutional and pooling layers provides an increased accuracy of 1.10% (model O) compared to the same settings without any dropout (model P). Previously, the classification results showed a lot of bias within the baseline model with a considerable offset between precision and recall due to a heavy bias towards predicting the benign class. However, through adding dropout layers, the inherent biases in the model are reduced, resulting in a reduction in this disparity to only 1%, and more importantly, a greater ability in classifying malignant samples. Ultimately, for tasks like cancer detection, it is more important that a model is sensitive to the malignant class to avoid incorrectly diagnosing patients that have a malignant tumour as having a benign tumour.

## Conclusion

This study saw the design and implementation of a deep learning pipeline capable of performing mammography classification for breast cancer detection through various deep learning techniques. After investigating a wide array of techniques using a divide and conquer approach, the greatest accuracy gain of 5.6% relative to the baseline (total accuracy of 67.4%) was obtained using transfer learning techniques (Fig 8, Model H). This model used ImageNet weights with MobileNetV2 and randomly instantiated weights for the custom MLP layers, coupled with class weights for balancing the dataset and other deep learning-based techniques. Additional results for this model, including a normalised confusion matrix (S1 Fig), AUC/ROC (S2 Fig) and additional metrics (S1 Table), can be found in the Supporting Information section.

Further, separating samples between masses and calcifications also yielded increased accuracies compared to the benchmark (64.35% and 66.67% respectively) when using VGG19 as a base model (Fig 8, models L and M). However, other techniques did not behave as expected and resulted in poor accuracies, such as separating samples between masses and calcifications with MobileNetV2 or using larger input images with extra convolutional and pooling layers to learn lower-level features without any image pre-processing steps such as histogram equalisation and Gaussian filtering.

Although other papers achieve more performance in their final model, notably the ones explored in the Context Review, the ultimate goal of this paper is to serve as a guide for mammography classification tasks that use deep learning techniques through the divide and conquer approach rather than aiming to develop a single model with superior performance. It is worth noting that the combination of deep learning techniques used by model H outperforms studies that use different machine learning techniques on the same CBIS-DDSM dataset such as Sarosa et al.’s SVM model with GLCM features [64].

### Limitations

A known limitation concerning all breast cancer detection systems lies with the data itself, as the most widely used datasets of mammograms (e.g. DDSM) contain data that mainly originates from white females located in North America, which naturally introduces bias to the model learning this data [16]. Different body types linked to the geographic location of the patients used to create these databases can have a direct impact on the mammograms themselves and not generalise to females from other cultures. For example, a recent study with 53,000 North American females showed how diets that include dairy milk consumption might increase the risk of breast cancer by a maximum of 80% based on the consumption [65]. This means that if these deep learning algorithms were implemented in clinics outside western countries, they might not generalise well to other body morphologies (e.g. due to different diets based on the geolocation’s culture). This limitation could be resolved by collecting more varied data from multiple locations around the world, not just a single region, which would also help the deep learning algorithms used as more data is always more beneficial [66].

Another limitation in terms of the detection system’s usability is the confidence of the predictions. Indeed, when given new test samples, the model predicts a class label, e.g. benign or malignant. However, these do not indicate the prediction’s confidence, as it can be anywhere between the decision boundary’s limit (not confident) and far from the decision boundary (confident). Therefore, from a clinical point of view, it is hard to make a decision based on the predictions made by a system similar to this one. Ideally, a probability-based confidence metric would be coupled with the predictions to motivate the next step after the diagnosis. For example, if the confidence of a malignant tumour is high (e.g. 99%), then breast-conserving surgery or chemotherapy can be recommended, whereas if the confidence is low (e.g. low 50%s), then further screening tests can be recommended instead.

### Future work

The main area of work that requires improvements is the mammogram imagery pre-processing as it is often an area where significant performance gains can be found [12] by using techniques such as Global Contrast Normalisation (GCN), local contrast normalisation, and Otsu’s threshold segmentation. Artefacts such as tags on the x-rays and black backgrounds should all be removed using computer vision techniques to avoid the CNN learning irrelevant features. A couple of ways to do so could involve cropping the image directly around the breast area or introducing bounding boxes for ROIs. Those improvements, accompanied with providing the degree of certainty with regards to the binary classification, would increase the clarity of the predictions for clinical practitioners and allow for improved human cross-validation and/or investigation of the pipeline’s output.

Another area where improvements can be made is the fine-tuning to extract better performance on the datasets and avoid overfitting. With the data pre-processing mentioned above, images would be smaller (e.g. no redundant dark background), which would in turn allow for quicker fitting and inference runtimes revealed, allowing hyperparameter fine-tuning algorithms like grid search or Bayesian optimisation to explore more combinations of configurations in order to unlock better solutions.

Finally, future research could look into applying the results found in this paper on other mammography datasets, such as the recently released Chinese Mammography Database (CMMD) dataset [24] and other datasets that introduce greater heterogeneity across its samples. This would improve generalisability with regards to imaging and cross-continental variation in body morphologies. Expanding the classification task from a binary to multi-class one by using different datasets would also help make the pipeline more versatile as it could not only be able to classify benign and malignant cases, but also normal cases with no masses or calcifications.

## Supporting information

S1 Fig Normalised confusion matrix. Normalised confusion matrix of the predictions made by the model achieving the highest accuracy (Fig 8, Model H) on the test set.
(PNG)

S2 Fig Area Under Curve Receiver Operator Characteristic (AUC/ROC). Area Under Curve Receiver Operator Characteristic (AUC/ROC) of the model achieving the highest accuracy (Fig 8, Model H) on the test set.
(PNG)

S1 Table Additional classification metrics. Precision, recall, F1 score and overall accuracy of the model achieving the highest accuracy (Fig 8, Model H) on the test set.
(XLSX)
