# Supplementary figures and images for: A divide and conquer approach to maximise deep learning mammography classification accuracies
Source: PLoS One. 2023 May 26;18(5):e0280841. doi: 10.1371/journal.pone.0280841 (PMC10218746; doi:10.1371/journal.pone.0280841)

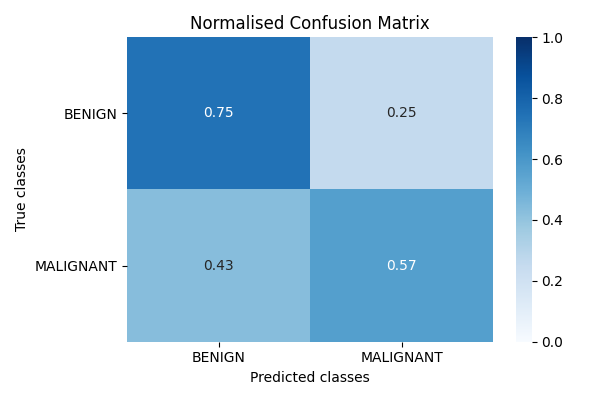

Supplement: S1 Fig — Normalised confusion matrix of the predictions made by the model achieving the highest accuracy (Fig 8, Model H) on the test set. (PNG) [file pone.0280841.s001.png]

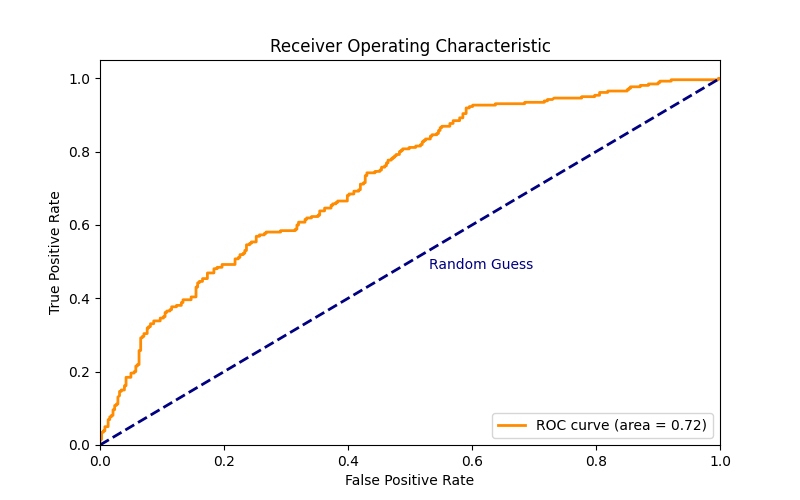

Supplement: S2 Fig — Area Under Curve Receiver Operator Characteristic (AUC/ROC) of the model achieving the highest accuracy (Fig 8, Model H) on the test set. (PNG) [file pone.0280841.s002.png]
